# Characterization of PSOP26 as an ookinete surface antigen with improved transmission-blocking activity when fused with PSOP25

**DOI:** 10.1186/s13071-022-05294-8

**Published:** 2022-05-23

**Authors:** Peng-peng Wang, Xuefeng Jiang, Jie Bai, Fan Yang, Xinxin Yu, Yudi Wu, Wenqi Zheng, Yongzhe Zhang, Liwang Cui, Fei Liu, Xiaotong Zhu, Yaming Cao

**Affiliations:** 1 Department of Immunology, College of Basic Medical Sciences, China Medical University, Shenyang, 110122 Liaoning China; 2 Department of Laboratory Medicine, Shengjing Hospital of China Medical University, Shenyang, 110004 China; 3 Department of Clinical Laboratory, Affiliated Hospital of Inner Mongolian Medical University, Inner Mongolia, Huhhot, 150000 China; 4 Department of Pathogen Biology, College of Basic Medical Sciences, China Medical University, Shenyang, 110122 Liaoning China; 5 Department of Nephrology, Shengjing Hospital of China Medical University, Shenyang, 110004 Liaoning China; 6 Department of Internal Medicine, Morsani College of Medicine, University of South Florida, 3720 Spectrum Boulevard, Suite 304, Tampa, FL 33612-9415 USA

**Keywords:** *Plasmodium berghei*, Gamete, Ookinete, Fertilization, Transmission-blocking

## Abstract

**Background:**

The *Plasmodium* zygote-to-ookinete developmental transition is an essential step for establishing an infection in the mosquito vector, and antigens expressed during this stage are potential targets for transmission-blocking vaccines (TBVs). The secreted ookinete protein 26 (PSOP26) is a newly identified ookinete surface protein. The anti-PSOP26 serum has moderate transmission-blocking activity, indicating the benefit of further investigating this protein as a target for TBVs.

**Methods:**

The function of *psop26* was analyzed by targeted gene disruption. A chimeric PSOP25-PSOP26 protein was expressed in the *Escherichia coli* system. The PSOP25-PSOP26 fusion protein, along with mixed (PSOP25 + PSOP26) or single proteins (PSOP26 or PSOP25), were used for the immunization of mice. The antibody titers and immunogenicity of individual sera were analyzed by enzyme-linked immunoassay (ELISA), indirect immunofluorescence assay (IFA), and Western blot. The transmission-blocking activity of sera from different immunization schemes was assessed using in vitro and in vivo assays.

**Results:**

PSOP26 is a surface protein expressed in *Plasmodium* gametes and ookinetes. The protein is dispensable for asexual blood-stage development, gametogenesis, and zygote formation, but is essential for the zygote-to-ookinete developmental transition. Specifically, both the prevalence of infections and oocyst densities were decreased in mosquitoes fed on *psop26*-null mutants. Mixtures of individual PSOP25 and PSOP26 fragments (PSOP25 + PSOP26), as well as chimeras (PSOP25-PSOP26), elicited high antibody levels in mice, with no immunological interference. Antisera against the mixed and fusion proteins elicited higher transmission-reducing activity (TRA) than antisera against the single PSOP26 antigen, but comparable to antisera against PSOP25 antigen alone.

**Conclusions:**

PSOP26 plays a critical role in the zygote-to-ookinete developmental transition. PSOP25 is a promising TBV candidate that could be used alone to target the ookinete stage.

**Supplementary Information:**

The online version contains supplementary material available at 10.1186/s13071-022-05294-8.

## Background

Malaria deaths in Africa have decreased by 44% since 2000, but progress has slowed in recent years (WHO, 2020). This may be due to factors such as shortfalls in funding, resistance to widely used insecticides and anti-malaria drugs, and region-specific inadequacies in health systems [1]. Therefore, new technologies to eliminate malaria from all areas are urgently needed, which can be developed in part by a better understanding of disease transmission.

The parasite sexual stages mediate transmission to mosquitoes, and thereby the spread of malaria. Gametes fertilize in the mosquito midgut to form zygotes, which develop into motile invasive ookinetes that traverse the midgut epithelium to differentiate into oocysts containing hundreds of sporozoites. Following release from oocysts, sporozoites pass into the salivary glands, in wait for transmission to a new vertebrate host during the next blood meal by the infected mosquito. The zygote-to-ookinete developmental transition and the following oocyst development are essential for the malaria parasite to complete its transmission life cycle. Antigens expressed during these key phases have been analyzed extensively; such as the major ookinete surface proteins P25 and P28 [2], secreted ookinete adhesive protein (SOAP) [3], the *Plasmodium* perforin-like protein (PPLP) family [4], and the putative secreted ookinete proteins (PSOPs) [5, 6]. PSOPs have been implicated to play roles in the zygote-to-ookinete and ookinete-to-oocyst developmental transitions; for example, *psop2*- and *psop25*-null mutants are defective for in vivo ookinete development, PIMMS2/57/22 promotes ookinete invasion [7, 8], *pimms1/57/22*-null mutants are defective for the ookinete-to-oocyst transition, and *pimms22*-null sporozoites are unable to initiate transmission [8]. Together, these results highlight the role of PSOPs in malaria parasite transmission.

Vaccines designed to induce antibodies that interrupt malaria transmission by targeting sexual stages are referred to as transmission-blocking vaccines (TBVs). Instead of providing direct protection against infection or disease in the human host, these vaccines are intended to prevent parasite transmission from an infected human to a feeding mosquito. At present, a broad range of antibodies against sexual-stage proteins have been screened for transmission-blocking activity (TBA). Among them, P48/45, P230, and P25/28 are leading candidates for vaccines preventing malaria parasite transmission to mosquitoes [9–11]. Pfs230 and Pfs48/45 are pre-fertilization TBV candidates, while the P25/28 pair are post-fertilization TBV candidates which are expressed on the surface of ookinetes. The P25/28 antigens exhibit promising TBA in animal studies, but when formulated with alum as an adjuvant, both *Plasmodium falciparum* and *Plasmodium vivax* P25 showed poor immunogenicity and no transmission-blocking effect in a human trial [12, 13]. With the use of Montanide ISA 51 as an adjuvant, Pfs25 and Pvs25 were able to induce transmission-blocking immunity in humans, but the higher formulated dose used for immunization was associated with the risk of systemic adverse events, such as erythema nodosum and leukopenia [14]. A clinical trial for Pfs25-IMX313 proved its safety and tolerance for malaria-naïve adults, but this vaccine exhibits weak TBA [15]. Another clinical trial carried out in the United States and Mali using Pfs25-ExoProtein A (EPA) formulated with Alhydrogel™ showed that four doses are required to generate antibodies that possess effective TBA, and the protective antibody titers decreased rapidly after the final immunization [16, 17]. Further, Pfs25-EPA combined with Pfs230D1-EPA did not increase activity over Pfs230D1-EPA alone [18]. These studies highlight that alternative antigens or combinations should be assessed to improve TBA.

Several studies have shown that TBA can be improved by taking advantage of the synergy between two antigens, such as Pfs25 and Pfs28 [19]. However, mixed or fusion antigens carry the risk of immunological interference between antigens, which could lead to a reduced immune response to one or both of the components. Such a case was observed for MSP1 and AMA1, in which a lower antibody titer of AMA1 was observed as compared to AMA1 administered alone [20]. In another example, although dual-antigen vaccines of Pfs25 fused or mixed with Pfs28 or Pfs230C did not cause immunological interference, they could not improve the transmission-reducing activity (TRA) compared to mono-antigen vaccines [21]. Collectively, these findings suggest that the TBA may be dependent on specific antigens used in the combinations.

Our previous study revealed two ookinete surface proteins, PSOP25 and PSOP26, as new targets for transmission-blocking interventions. Mice immunized with either recombinant PSOP25 or PSOP26 resulted in sera which in mosquito feeding assays provoked a moderate reduction of oocyst density (60.0–70.7%) and prevalence of infected mosquitoes (20.1–37.4%) [6, 22]. Since neither PSOP25 nor PSOP26 antigen alone could produce 100% TBA efficacy, functional mapping and testing of the combination of these two surface antigens to improve TBA is warranted. Thus, in this study, we characterized the function of PSOP26 protein during sexual-stage development and evaluated the fusion and mixed forms of PSOP26 and PSOP25 antigen compared to mono-antigens in terms of immunogenicity and TRA.

## Methods

### Mice, parasites, and mosquitoes

The *Plasmodium berghei* ANKA 2.34 line was used for genome editing. All parasite lines were maintained in 6-week-old female BALB/c mice (Beijing Animal Institute, Beijing, China). *Plasmodium berghei* schizonts, gametocytes, zygotes, and ookinetes were cultured and purified as described previously [22]. *Anopheles stephensi* mosquitoes of the Hor strain were fed on a 10% (w/v) glucose solution, maintained at 25 °C with 50–80% humidity, and a 12/12 h light/dark cycle. All animal procedures were performed in accordance with the welfare and ethical review standards of China Medical University.

### Quantitative real-time polymerase chain reaction (qRT-PCR)

To determine the transcription profile of the *psop26* gene (PlasmoDB ID: PBANKA_1457700), total RNA was isolated from purified *P. berghei* schizonts, gametocytes, and ookinetes parasites using an RNeasy Plus Universal Kit (Qiagen, Dusseldorf, Germany) followed by DNase I (Invitrogen, Waltham, MA, USA) treatment to remove genomic (gDNA). Complementary DNA (cDNA) was synthesized using a High-Capacity cDNA Reverse Transcription Kit (Thermo Fisher, Waltham, MA, USA). qRT-PCR analysis was conducted using PrimeScript™ RT Master Mix (Perfect Real Time, Thermo Fisher) and gene-specific primers according to the kit protocol. Gene expression of *psop26* was normalized against β-tubulin (PBANKA_120690) in *P. berghei* using the 2^−ΔΔCt^ method (see Additional file 1: Table S1 for primer sequences). Analysis was conducted using a 7500 Fast PCR System (Thermo Fisher).

### Generation of transgenic parasites

The strategy for generating transgenic parasite lines involved electroporation of gene target vectors into purified schizonts, followed by infection of mice by intravenous injection as described previously [23]. To tag the endogenous *psop26* with a 3 × HA tag, a 769-base-pair (bp) fragment [nucleotide (nt) positions 1554–2322 bp] of the *psop26* gene was amplified from *P. berghei* genomic DNA gDNA and ligated into the ApaI and SacII sites of the pL0034 plasmid as the 5′ homologous region (5R). Then, 526 bp of the *psop26* 3′ untranslated region (UTR) (nt + 1– + 526 bp) was amplified and inserted between the KpnI and EcoRI sites as the 3′ homologous region (3R), to yield the final plasmid pL0034-PSOP26-3 × HA. To disrupt the *psop26* gene, a ∆*psop26* transgenic parasite was generated using the PbGEM-325763 plasmid (kindly provided by PlasmoGEM; http://plasmogen.sanger.ac.uk/). The linearized plasmid (10 µg) was transfected into purified *P. berghei* schizonts using a Nucleofector II device (Lonza, Basel, Switzerland) and the Basic Parasite Nucleofector^®^ kit (Lonza). The transfected parasites were injected into BALB/c mice via the tail vein and selected using pyrimethamine (70 µg/ml, Sigma-Aldrich, St. Louis, MO, USA) in the drinking water. Parasite gDNAs were isolated from blood-stage parasites using a DNeasy Blood kit (Qiagen), and the correct integration of the 5′ and 3′ homologous regions was detected using genotype PCR analysis. All primers used in this study are listed in Additional file 1, Table S1. Parasite clones with targeted modifications were obtained after limiting dilution. At least two clones for each gene-modified parasite were used for phenotype analysis.

### Western blot analysis

For stage-specific protein expression assays, purified schizonts, gametocytes, zygotes, and ookinetes were treated with 0.15% saponin (Sigma) in phosphate-buffered saline (PBS) for 10 min on ice, followed by three washes with PBS containing 1× protease inhibitor cocktail (PBS-PI, Thermo Fisher) to remove hemoglobin. To detect the expression of recombinant proteins in transgenic parasites, mixed stages of wild-type (WT) and transgenic parasites were directly lysed using 0.15% saponin. Protein samples were boiled and separated by 10% sodium dodecyl sulfate–polyacrylamide gel electrophoresis (SDS-PAGE) under reducing conditions and transferred to polyvinylidene fluoride (PVDF) membranes (Bio-Rad, Hercules, CA, USA). The PVDF membranes were blocked with 5% skim milk and then probed using mouse anti-HA monoclonal antibody (mAb, 1:1000; Invitrogen, Carlsbad, CA) and anti-rHSP70 sera (1:2000; PlasmoDB ID: PBANKA_0711900, produced in our laboratory). After washing three times, the membranes were probed with horseradish peroxidase (HRP)-conjugated goat anti-mouse immunoglobulin G (IgG) antibodies (1:5000, Thermo Fisher). The blots were then detected using an ECL Western Blotting Kit (Pierce, Rockford, IL, USA).

### Indirect immunofluorescence assay (IFA)

The subcellular localization of PSOP26 and PSOP25 was analyzed by IFA as described previously [24]. The HA-tagged transgenic or WT *P. berghei* parasites were fixed with 4% paraformaldehyde and 0.0075% glutaraldehyde (Sigma) in PBS for 30 min at room temperature (RT). Cells were either permeabilized with 0.1% v/v Triton X-100 or directly processed without permeabilization. Then the parasites were neutralized with 0.1 mg/ml of sodium borohydride and blocked in 3% w/v BSA (diluted in PBS). Parasites were then stained with anti-HA mAb (1:500, Invitrogen) or antisera against PSOP26 or PSOP25. Parasites were co-labeled with rabbit antisera against PbMSP1 (1:500), Pbg377 (1:500), α-tubulin II (1:500), or PSOP25 (1:500) as stage-specific markers for schizonts, female gametocytes/gametes, male gametocytes/gametes, and zygotes/ookinetes, respectively. The non-permeabilized parasites were treated with 0.1% v/v Triton X-100 before labeling with these stage-specific markers. Alexa Fluor 488-conjugated goat anti-mouse IgG antibodies (1:500; Invitrogen) and Alexa Fluor 555-conjugated goat anti-rabbit IgG antibodies (1:500; Abcam, Cambridge, UK) were used as secondary antibodies. Parasite nuclei were stained with Hoechst 33258 (Invitrogen) at a final concentration of 5 μg/ml. Negative controls were HA-tagged ookinetes incubated with the secondary antibodies alone, or WT ookinetes incubated with the anti-HA mAb. The antibodies against PbMSP1, Pbg377, α-tubulin II, and PSOP25 were made in our laboratory [25]. Parasites were visualized using a Nikon C2 fluorescence confocal laser scanning microscope (Nikon, Tokyo, Japan).

### Phenotypic analysis of the *∆psop26* transgenic parasite

To determine the function of PSOP26 in the *P. berghei* life cycle, 5 × 10^6^ *P. berghei* wild-type (WT) or *∆psop26* infected red blood cells (iRBCs) were intravenously administered to mice pretreated with 6 mg/ml phenylhydrazine (PHZ). Parasitemia and mortality of the infected mice were monitored daily. To assess the function of parasite sexual-stage development, gametocytemias (mature gametocytes per 10^4^ RBCs) and gametocyte sex ratios (female to male gametocyte ratio) were counted at 3 days post-infection (dpi). Exflagellation centers and male–female gamete interactions were assessed as described previously [26]. Briefly, 10 μl of infected blood containing equal numbers of mature gametocytes was added to 40 μl ookinete medium (RPMI 1640, 50 mg/l penicillin, 50 mg/l streptomycin, 20% [v/v] FCS, 6 U/ml heparin, pH 8.0) for 15 min at 25 °C; then 1 μl of the culture mixture was smeared onto a glass slide (Matsunami Glass Ind., Ltd., Japan) and exflagellation centers (an exflagellating male gametocyte with more than four adhered RBCs) were counted using a light microscope. The male–female gamete interactions were calculated as male gametes attached to female gametes for more than 3 s in 10 fields within 20 min [27]. To count the number of macrogametes, 10 μl of infected blood was mixed with 90 μl ookinete medium for 15 min at 25 °C; then 0.5 μl of the mixture was placed on a slide, fixed, and labeled with anti-Pbs21 mAb (clone 13.1, 1:500, a gift from Dr. Hiroyuki Matsuoka from Jichi Medical University, Japan), and Pbs21-positive macrogametes were counted using a fluorescence microscope. The culture was subsequently incubated at 19 °C for 2 h and 24 h, labeled with anti-Pbs21 mAb (1:500), and the numbers of zygotes and ookinetes, respectively, in 0.5 μl of the cultures were counted using a fluorescence microscope. For oocyst quantification, at 3 dpi, WT or *∆psop26* parasite-infected mice were fed to starved female anopheline mosquitoes for 1 h. Ten days after feeding, ~ 30 fed mosquitoes from each group were dissected for counting the oocyst numbers per infected mosquitoes and to determine the prevalence and intensity of infection.

### Recombinant protein expression and immunization

To generate a chimeric PSOP25 and PSOP26 protein (PSOP25-PSOP26), gene fragments of PSOP25 [45–245 amino acids (aa)] and PSOP26 (50–254 aa) were fused with a flexible linker (GGGGS)_3_ between the two sequences by overlapping PCR using primers described in Additional file 1: Table S1. The PCR products were cloned into the vector pET-32a (+) (Novagen, Darmstadt, Germany). Recombinant proteins were expressed in *E. coli* Rosetta-gami B (DE3) cells under induction with 1 mM isopropyl β-d-1-thiogalactopyranoside (IPTG; Sigma) at 19 °C for 16 h. The His-tagged proteins were affinity purified using Ni–NTA His-Bind Superflow resin (Novagen), followed by dialysis in 0.1 M PBS at 4 °C overnight. Purified recombinant proteins were cleaved by enterokinases at 25 °C for 16 h, and further purified with Ni–NTA His-Bind Superflow resin. The final recombinant proteins were analyzed by 10% SDS-PAGE to determine purity.

For the generation of serum against recombinant PSOP25, PSOP26, PSOP25 + PSOP26, and PSOP25-PSOP26, five female BALB/c mice for each group were immunized subcutaneously with the emulsified product of recombinant protein (50 μg per mouse for PSOP25 and PSOP26, and PSOP25-PSOP26 groups; 50 µg PSOP25 + 50 µg PSOP26 per mice for the PSOP25 + PSOP26 group) and complete Freund’s adjuvant (Sigma). The immunizations were enhanced at weeks 2 and 4 with recombinant proteins emulsified in incomplete Freund’s adjuvant (Sigma). At 14 days after the final immunization, blood was collected by cardiac puncture and agglutinated at room temperature to obtain antisera, and stored at −80 °C for the subsequent trials.

### Enzyme-linked immunosorbent assay (ELISA)

Serum antibody titers were measured by ELISA as described previously [28]. The 96-well plates were coated with 5 μg/ml recombinant PSOP25 or PSOP26 in 0.05 M sodium carbonate buffer (pH 9.6) at 4 °C overnight. The plates were then washed three times with 200 μl PBS containing 0.02% Tween-20 (PBS-T) and blocked with 1% bovine serum albumin (Sigma) for 1 h at 37 °C. After washing an additional three times with PBS-T, 100 μl serial dilutions of antisera (from 1:200 to 1:25,600) were added to the 96-well plates and incubated at 37 °C for 2 h. After three washes, HRP-conjugated goat anti-mouse IgG antibodies (1:5000 dilutions) were added to the plates and incubated for 1 h at 37 °C. After six washes, 100 μl of tetramethylbenzidine (TMB, Sigma) was added and incubated in the dark for 5 min. The reaction was stopped with 50 μl of 1 mM H_2_SO_4_, and the absorbance at 490 nm was measured using a Bio-Rad ELISA microplate reader.

### Transmission blocking analysis

Polyclonal antibodies generated against the above recombinant proteins were utilized in transmission-blocking assays. For the in vitro assay, PHZ pretreated mice were infected intraperitoneally with 5 × 10^6^ *P. berghei* iRBC. At 3 dpi, parasitemias were determined, and 10 μl of blood was taken from appropriate hosts and added to 40 μl ookinete culture medium containing anti-PSOP26, PSOP25, PSOP25 + PSOP26, and PSOP25-PSOP26 sera or negative control mouse serum at final dilutions of 1:5 and 1:10. The exflagellation centers per field were quantified as described above. For ookinete formation analysis, ookinete cultures were incubated at 19 °C for 24 h and the number of matured ookinetes was determined by anti-Pbs mAb staining as described above. The ookinete conversion rates were calculated as the percentage of Pbs21-positive ookinete to Pbs21-positive macrogametes and ookinetes as described previously [29, 30].

For direct mosquito feeding assays (DFA), PHZ pretreated mice were infected with 5 × 10^6^ *P. berghei* iRBC as described above. At 3 dpi, 4-day-old *An. stephensi* mosquitoes pre-starved for 12 h were allowed to feed on infected mice for 1 h (~ 50 mosquitoes/mouse). The unfed mosquitoes were removed and the remaining mosquitoes were maintained at 19–22 °C and 50–80% relative humidity. At 12 days post-feeding,  ~ 30 mosquitoes in each group were dissected and stained with 0.5% Mercurochrome to count the number of oocysts per mosquito and assess the oocyst infection intensity and oocyst infection prevalence.

### Statistical analysis

All statistical analyses were performed using GraphPad Prism software 8.0. Parasitemias, gametocytemias, and gametocyte sex ratios were compared by Student’s *t*-test. The zygote and ookinete numbers between groups were compared using the Kruskal–Wallis test followed by Dunn’s multiple comparisons test. The Kaplan–Meier method was used to analyze the survival of mice. The prevalence of infection (proportion of infected mosquitoes) was analyzed by the Fisher’s exact test (with Bonferroni correction), while the oocyst density (oocyst number per midgut) was analyzed by the Mann–Whitney *U*-test. A *P*-value less than 0.05 was considered statistically significant.

## Results

### PSOP26::HA localizes to the surface of gamete and ookinete

**Fig. 1:**
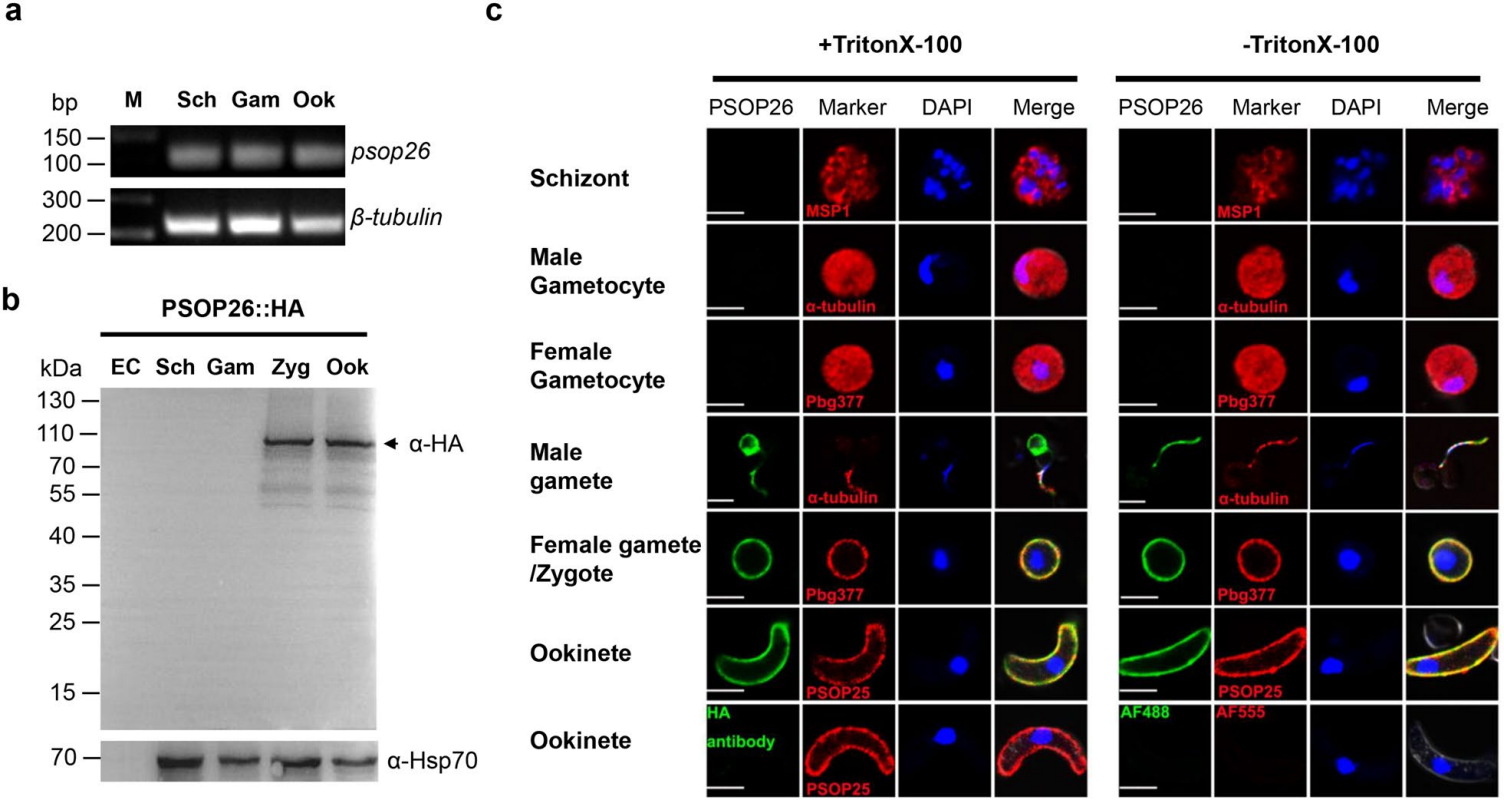
PSOP26 transcript levels, protein expression, and localization on the surface of gametes and ookinetes in *P. berghei*. **a** PSOP26 RT-PCR transcription profiles. Real-time RT-PCR analysis of the expression profile of *psop26* transcripts in schizonts (Sch), non-activated gametocytes (Gam), and ookinetes (Ook). The β-tubulin gene, which is constitutively expressed at all three stages, was used as a loading control. **b** Expression of PSOP26::HA during parasite development. Western blot analysis of proteins extracted from purified *P. berghei* schizonts (Sch), gametocytes (Gam), zygotes (Zyg), and ookinetes (Ook) of PSOP26-HA parasites. Reduced lysates were separated by SDS-PAGE, transferred to a PVDF membrane, and labeled with anti-HA antibody and anti-Hsp70 sera (loading control). Non-infected erythrocytes (EC) were used as a negative control. **c** Immunofluorescence assays of Triton X-100 permeabilized (+Triton X-100, left) or non-permeabilized (−Triton X-100, right) *P. berghei* ring, trophozoite, schizont, gametocytes, gametes, zygotes, and ookinetes. Stained with α-HA monoclonal antibody (green), α-msp1 (merozoite surface protein, red) and/or α-tubulin II (male gametocyte/gamete protein, red), and/or α-Pbg377 (female gametocyte/gamete protein, red), and/or α-PSOP25 (ookinete surface protein). DNA was stained with DAPI. Only the secondary antibodies or WT ookinetes incubated with the α-HA mAb were used as a negative control (AF488, Alexa Fluor 488, AF555, and Alexa Fluor 555). Merge denotes Alexa Flour 488 + Alexa Flour 555 + DAPI. Scale bars correspond to 5 μm

*Psop26* transcripts were detected in schizonts, non-activated gametocytes, and ookinetes using real-time RT-PCR analysis (Fig. 1a). To assess protein expression, we targeted *psop26* for in situ 3 × HA-tagging by double-crossover homologous recombination of a plasmid encoding a human dihydrofolate reductase:yeast cytosine deaminase and uridyl-phosphoribosyltransferase (*hDHFR*:*yFCU*) selectable marker (Additional file 2: Fig. S1a). PCR genotypic analysis was used to confirm successful modification of the endogenous genomic locus (Additional file 2: Fig. S1b). Despite the presence of transcripts in schizonts and non-activated gametocytes, the expression of the PSOP26::HA fusion protein was only detectable in zygotes and ookinetes; indicating that *psop26* messenger RNA (mRNA) translation starts after gametocyte activation (Fig. 1b and Additional file 3: Fig. S2). Examination of PSOP26::HA fusion protein by immunofluorescence assays showed parasite plasma membrane localization of PSOP26::HA protein in male gametes. The PSOP26::HA fusion protein was detectable both with and without Triton X-100 permeabilization; and was co-expressed with α-tubulin II (male gamete marker), Pbg377 (female gamete marker), and ookinete surface protein PSOP25 (Fig. 1c). Together, these data strongly support that PSOP26 is a membrane-associated protein in gametes and ookinetes.

### PSOP26 is required for the zygote-to-ookinete developmental transition

**Fig. 2:**
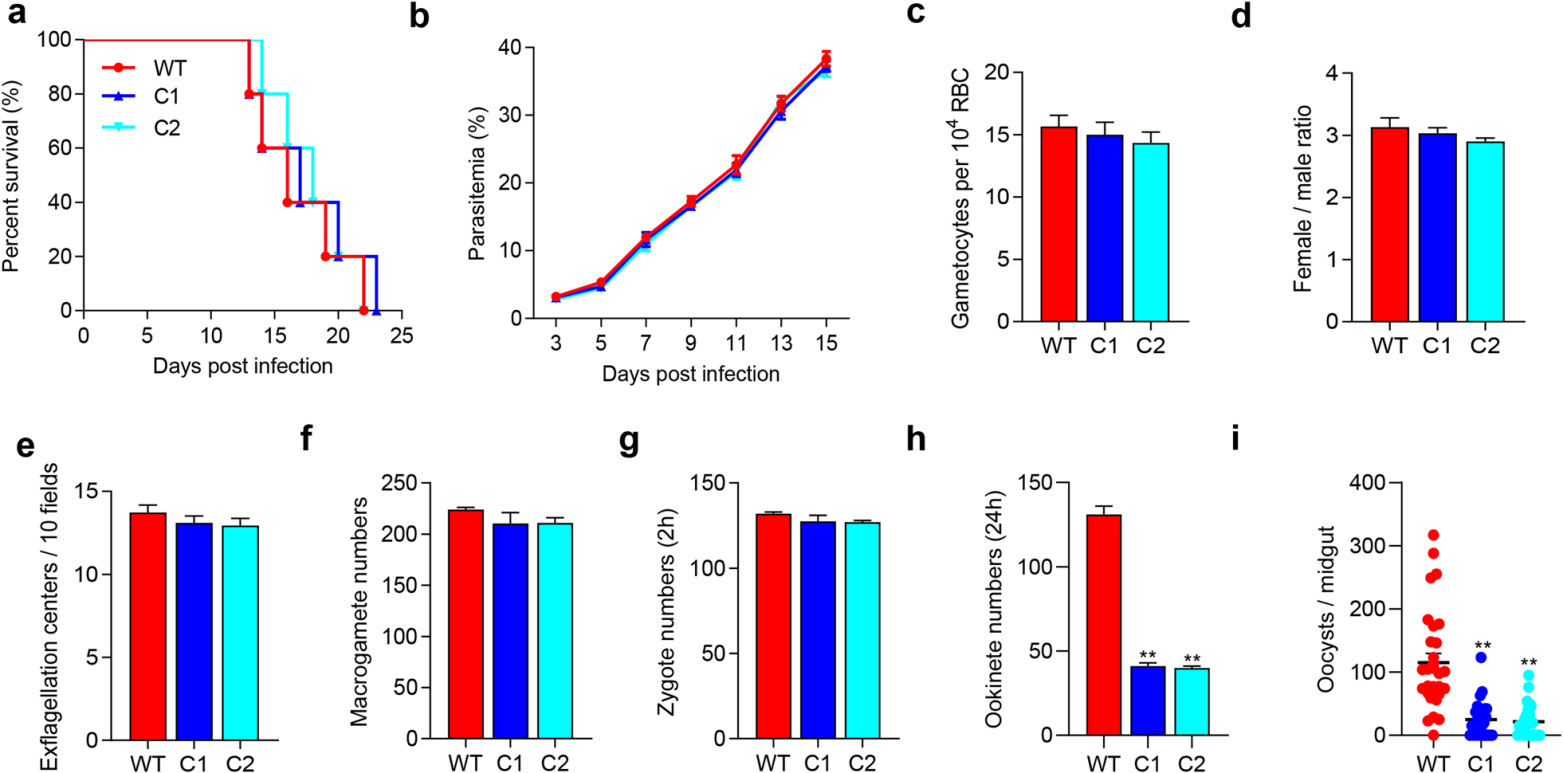
Phenotypic analyses of *∆psop26* mutants in asexual and sexual stages. Mice were injected with WT or *∆psop26* parasites clone 1 (C1) and clone 2 (C2). **a** Survival rates of mice. **b** Parasitemia was monitored by Giemsa smears after day 3 infection. **c** Gametocytemia (mature gametocytes per 10^4^ RBCs) on day 3 post-infection. **d** Gametocyte sex ratios (female: male) on day 3 post-infection with different parasite lines. Error bars indicate mean ± SEM (standard error of the mean) (*n* = 3). Data in the phenotypic analysis of *pbs54* and *psop26* are representative of three independent experiments. **e** Exflagellation centers in 10 fields at × 100 magnification with equal number of mature male gametocytes. **f** Female gamete numbers in 0.5 µl of ookinete culture at 15 min post-activation. **g** Zygote numbers formed at 2 h during in vitro ookinete culture. **h** Ookinete numbers cultured in vitro for 24 h. **i** Oocyst numbers per midgut in mosquitoes 12 days after feeding on WT, *∆pbs54*, or *∆psop26* parasite-infected mice. **P < 0.01 (Mann–Whitney *U*-test). Error bars indicate mean ± SEM (*n* = 3). Data in the phenotypic analysis of *psop26* are representative of three independent experiments

To identify the biological function of the PSOP26 protein, we generated two independent gene knockout mutants, designated Δ*psop26* clones C1 and C2. Integration of the disruption cassette (*hDHFR*:*yFCU*) was confirmed by diagnostic PCR (Additional file 2: Fig. S1c, d). Western blot analysis of total protein extracts prepared from purified *P. berghei* (WT) and Δ*psop26* ookinetes using anti-PSOP26 sera revealed a band of ~ 90.8 kDa in WT strain parasites, corresponding to the predicted molecular weight of endogenous PSOP26 protein (Additional file 2: Fig. S1e). This band was absent from Δ*psop26* ookinetes, indicating successful deletion of the *psop26* gene (Additional file 2: Fig. S1e). The absence of PSOP26 protein did not affect asexual blood-stage development, gametocytemia, or male-to-female gamete ratios; and the capability of Δ*psop26* gametocytes to produce exflagellation centers and macrogametes was comparable to that of the WT line (Fig. 2a–f). The number of zygotes formed remained unchanged, but the number of mature ookinete was reduced by 69% after 24 h in vitro culture, compared with WT parasites, suggesting that *psop26* deletion affected the zygote-to-ookinete developmental transition (Fig. 2g, h). Following a mosquito blood meal, mosquitoes fed on mice infected with the Δ*psop26* parasites exhibited 66% prevalence of infection, whereas the control group was 97%, a decrease of 31% (Table 1). Moreover, the mean number of oocysts per midgut was 23 in mosquitoes fed on *Δpsop26*-infected mice, compared with 115 in control mosquitoes, which indicates an 80% reduction (*P* < 0.01; Fig. 2i).

**Table 1:** Prevalence and oocyst density in mosquitoes fed on WT and *Δpsop26* parasites

Parasites	Infected/dissected	Infection prevalence^a^ (%)	Reduction in prevalence^b^ (%)	Oocyst density mean (range)^c^	Reduction in oocyst density^d^ (%)
WT	29/30	96.7		115.2 (0–317)	
*Δpsop26-C1*	19/29	65.5	31.2	24.9 (0–123)	78.4
*Δpsop26-C2*	20/30	66.7	30	21.4 (0–95)	81.4

^a^The infection prevalence was calculated based on the number of mosquitoes with oocysts/total mosquitoes dissected in each group × 100%

^b^The percentage reduction of prevalence was calculated as % mean prevalence_WT_ − % mean prevalence_*Δpsop26*_

^c^Mean number and range of oocysts per mosquito midgut

^d^The percentage reduction in oocyst density was calculated as (mean oocyst density_WT_ − mean oocyst density_*Δpsop26*_)/mean oocyst density_WT_ × 100%

### The chimeric PSOP25-PSOP26 protein is immunogenic

**Fig. 3:**
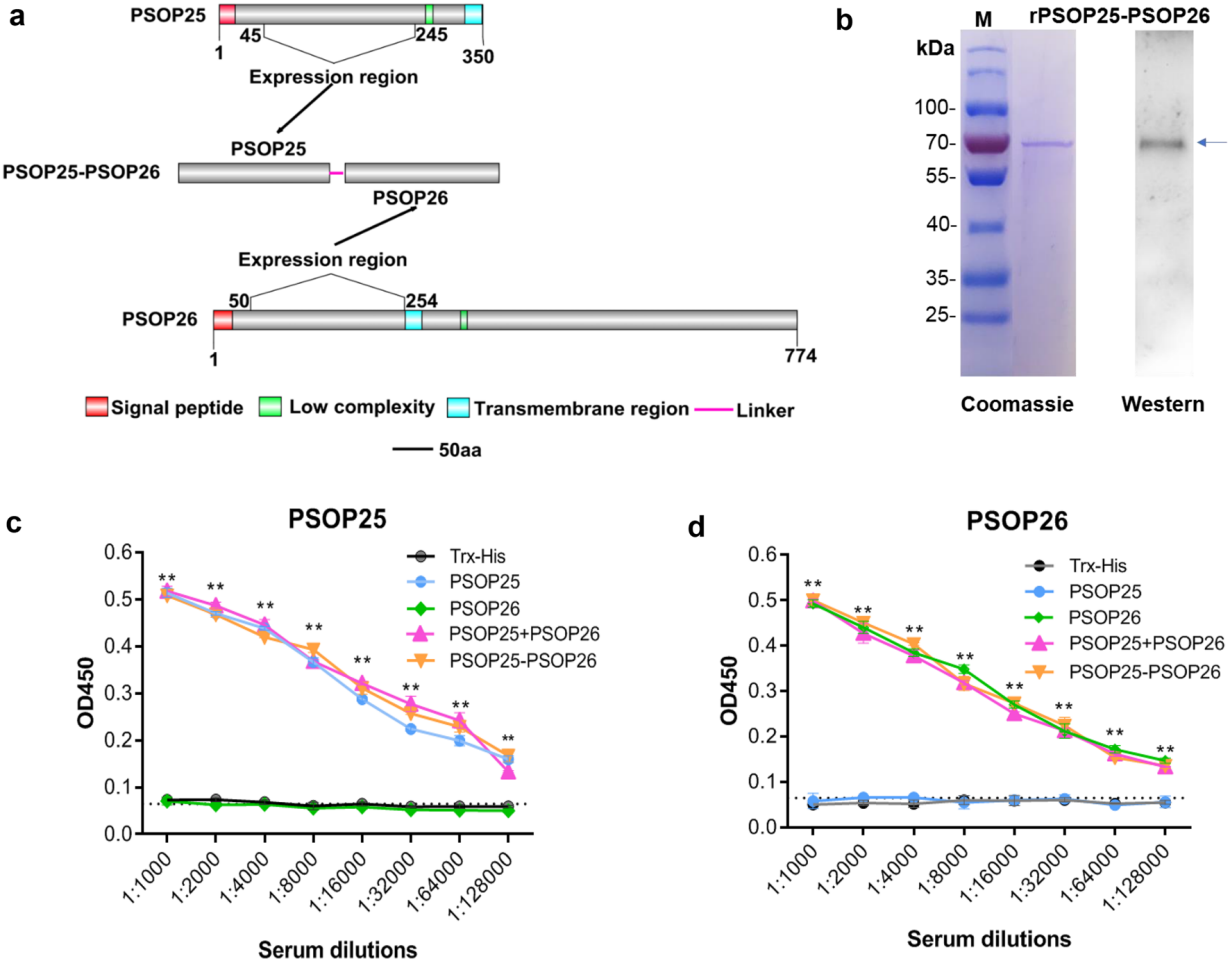
Purification of recombinant cPSOP25/26 protein and antibody response in mice immunized with rPSOP25, rPSOP26, rPSOP25 + PSOP26, and PSOP25-PSOP26. **a** Diagram illustrating the expressed regions of the PSOP26, PSOP25, and chimeric PSOP25-PSOP26. The signal peptide (red box), low complexity (green box), and transmembrane region (blue box) are highlighted. The pink line denotes the linker. **b** Purified recombinant PSOP25-PSOP26 (indicated by an arrow) was separated on a 10% SDS-PAGE gel and stained with Coomassie blue (left) and probed with anti-His mAb on a Western blot (right). BALB/c mice (*n* = 10) were immunized three times with Trx-His tag (immunization control) and recombinant proteins (rPSOP25, rPSOP26, rPSOP25 + PSOP26, and rPSOP25-PSOP26). Total IgG titers after final immunization were measured by ELISA analysis coated with recombinant PSOP25 (**c**) and recombinant PSOP26 (**d**) polypeptides after removal of the Trx tag. Error bars indicate standard deviation. **P < 0.01 indicates a significant difference between the immunization and control groups (Student’s *t*-test)

We previously reported that anti-PSOP26 sera, which recognizes a 242-aa fragment of PSOP26 (50–245 aa), blocked or reduced parasite transmission in DFA [22]. Thus, to investigate the potential benefit of combining sera specific to PSOP25 and PSOP26, to represent a combination vaccine, a chimeric construct within a pET-32a plasmid was designed containing the Asp50-Gln245 fragment of PSOP26 fused to the Met45-Glu245 fragment of PSOP25 using a flexible linker sequence (GGGGS)_3_. The chimeric PSOP25-PSOP26 protein (PSOP25-PSOP26) was induced in *E. coli* by 1 mM IPTG at 19 °C overnight, and purified using Ni–NTA chromatography (Fig. 3a). The expression of the recombinant protein was detected using an anti-His tag mAb, showing that the purified recombinant PSOP25-PSOP26 was approximately 70 kDa, consistent with the predicted combined molecular weight (Fig. 3b). To allow for better control in TBA analysis, recombinant PSOP25, PSOP26, and Trx-His proteins were expressed using the pET-32a plasmid (data not shown). The purified recombinant PSOP26 alone (PSOP26), mixed/fused with PSOP25 (PSOP25 + PSOP26 and PSOP25-PSOP26), or PSOP25 alone (PSOP25) was used to immunize mice to raise polyclonal antibodies. As expected, immunization with individual antigens yielded only antibodies specific for the respective antigens used for immunization. The antibody titers against PSOP25 induced by recombinant PSOP25 alone or mixed/fused with PSOP26 were similar as determined in ELISA analysis using immune serum collected 2 weeks after the final immunization (Fig. 3c). Similarly, the antibody titers were comparable against PSOP26 induced by recombinant PSOP26 alone, mixed, or fused with PSOP25 (Fig. 3d); indicating that no immune interference occurred between the two antigens.

### Reactivity of the antisera against mixed or fused PSOP25/PSOP26 with the native parasite proteins

**Fig. 4:**
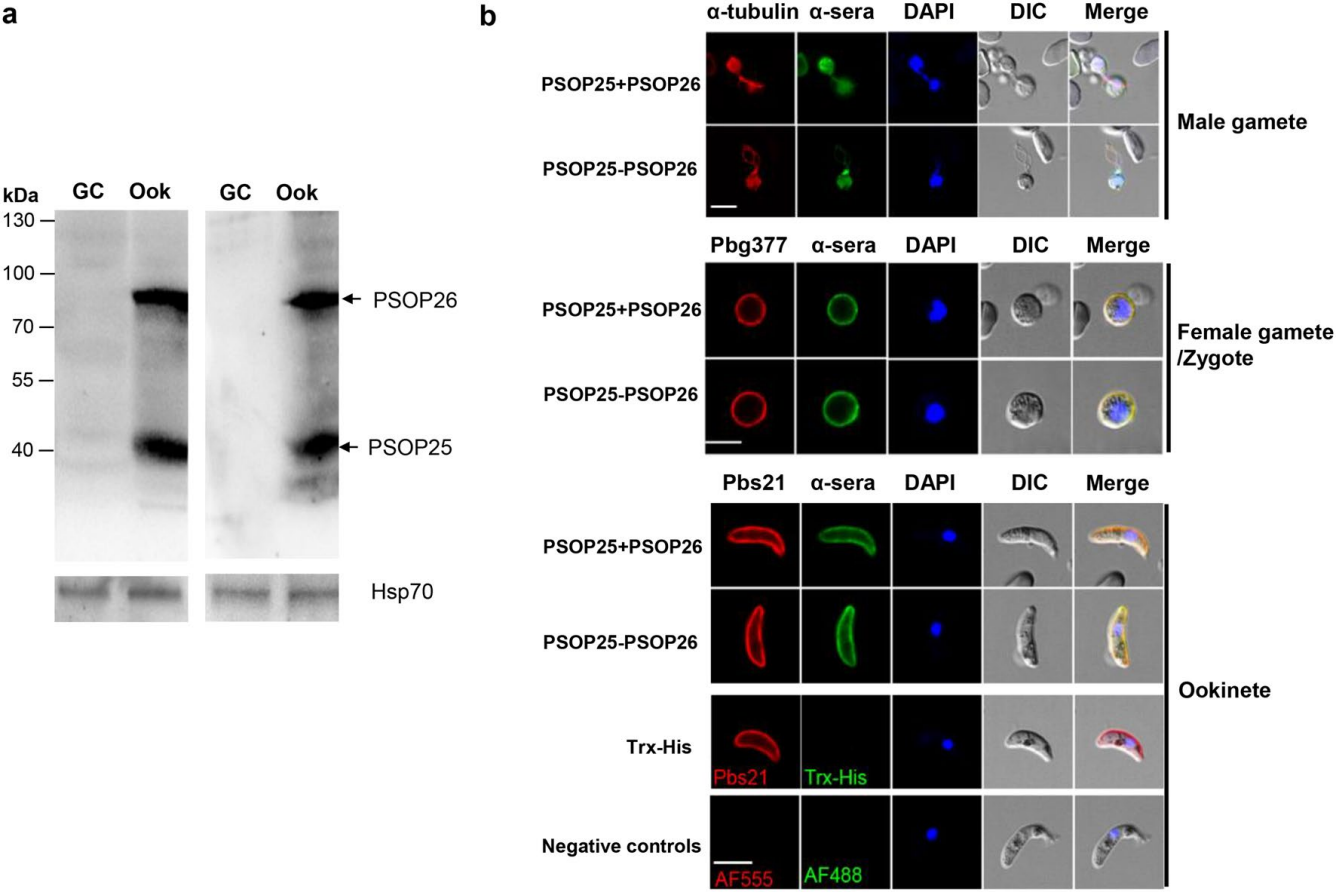
Reactivity of vaccine-induced antisera to native parasite antigen. **a** Western blot analysis under reducing conditions of gametocyte (GC) and ookinete (Ook) parasite lysates using anti-PSOP26 + PSOP26 sera (left) and anti-PSOP25-PSOP26 sera. PSOP25 and PSOP26 protein bands are indicated with arrows. Equal loading was estimated using anti-rHsp70 sera. **b** Indirect immunofluorescence analysis using the bivalent immune sera. Parasites of different developmental stages were fixed, permeabilized with 0.1% Triton X-100, and stained with antisera against PSOP25 + PSOP26 (left) and PSOP25-PSOP26 (right) (1:200) as the primary antibodies (green). The parasites were also co-labeled with antibodies against the markers for different stages (red), including α-tubulin (α) for male gametocytes/gametes, P47 for female gametocytes, Pbs21 for zygotes and ookinetes, and SET for the nucleus of gametocytes. Alexa Fluor 488-conjugated goat anti-mouse IgG antibodies and Alexa Fluor 555-conjugated goat anti-rabbit IgG antibodies were used as the secondary antibodies. The sera from mice receiving Trx-His recombinant proteins were included as negative controls. Antibody binding was detected by Alexa Fluor 555-conjugated goat anti-rabbit IgG (red) (lane 1), and Alexa Fluor 488-conjugated goat anti-mouse IgG (green) (lane 2). DNA was stained with DAPI (blue) (lane 3). DIC images (lane 4), and merged view (lane 5) are shown. Scale bar, 5 µm

Western blot analysis was performed using lysates from ookinetes to verify that the antisera produced against the antigen mixture or chimera reacted with parasite native proteins. Antisera against both mixed (PSOP25 + PSOP26) and fused (PSOP25-PSOP26) recombinant proteins recognized bands of 40 and 91 kDa in ookinete lysates, corresponding to PSOP25 and PSOP26 proteins, respectively (Fig. 4a). Next, we tested the ability of anti-PSOP25-PSOP26 sera and anti-PSOP25 + PSOP26 sera to recognize PSOP25 and/or PSOP26 expressed in different sexual stages of the parasite. The anti-PSOP25 sera and anti-PSOP26 sera were used as controls to ensure PSOP25 or PSOP26 expression. Both antisera against the mixed and fused antigen reacted with male gametes, female gametes/zygotes, and ookinetes (Fig. 4b). Comparable to the IFA results of the PSOP26-HA fusion protein, anti-PSOP25-PSOP26 and anti-PSOP25 + PSOP26 sera bound exflagellating male gametes, female gamete/zygotes, and ookinetes (Fig. 4b). Strong fluorescence signals were detected at the plasma membranes of female gamete/zygotes and ookinetes (Fig. 4b). Negative controls performed with antibodies against Trx-His or with the secondary antibodies alone did not react with these sexual stages (Fig. 4b). Collectively, antisera against the mixed or fused antigens from the pre- and post-fertilization stages were able to recognize the respective proteins expressed during the gamete to ookinete development.

### PSOP25 and PSOP26 fusion proteins elicit strong transmission-blocking antibody responses against *P. berghei*

**Fig. 5:**
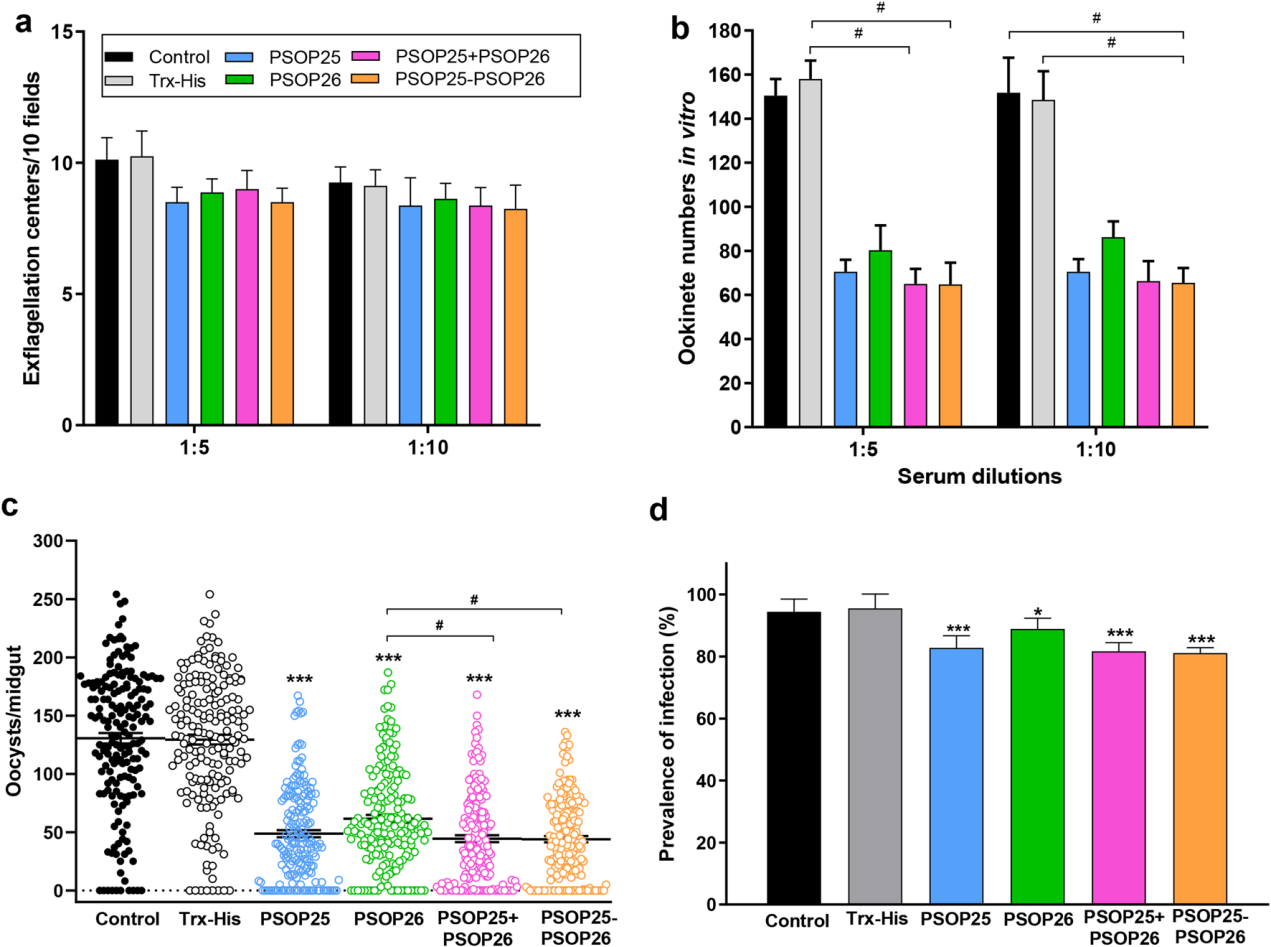
Transmission-blocking activity of the antisera assessed in vitro and in vivo. **a** Exflagellation centers per 10 fields. *Plasmodium berghei*-infected blood collected at 3 dpi was incubated with the respective control and immune sera at dilutions of 1:5 and 1:10. **b** Ookinete formation. The ookinete numbers after 24 h were stained with Pbs21 mAb and counted using a fluorescence microscope. The data were representative of three independent experiments. **c** Midgut oocyst numbers in mosquitoes fed on relative recombinant protein-immunized mice. The results were collected from three mice in each immunization group and two separate experiments (*n* = 180). Data points represent midgut oocyst numbers in individual mosquitoes. Horizontal bars indicate the mean number of oocysts per midgut. **d** Mosquito infection prevalence was calculated at 12 days after the blood meal. Data points represent the prevalence of infection in mosquitoes from three mice per group and in two separate experiments. Error bars indicate mean ± SD. *P < 0.05 and ***P < 0.001 represent the significant difference between the respective immunization group and the Trx-His control group. ^#^*P* < 0.05 ^#^ represents a significant difference between two immunization groups

The biological activity of specific antisera was assessed in a TBA assay as described in the methodology section. Although expressed on the surface of male gamete flagella, neither anti-PSOP25-PSOP26 sera nor anti-PSOP25 + PSOP26 sera supplements could inhibit male gametocyte exflagellation; suggesting that PSOP25 and PSOP26 do not play essential roles in male gametogenesis (Fig. 5a). Ookinete cultures supplemented with pooled immune sera raised against PSOP25, PSOP26, PSOP25 + PSOP26, or PSOP25-PSOP26 reduced ookinete numbers by 55%, 49%, 59%, and 59% at 1:5 dilutions, compared to the Trx-His group, respectively (Fig. 5b). The ookinete numbers of the cultures supplied with antisera against PSOP25, PSOP26, PSOP25 + PSOP26, or PSOP25-PSOP25 at 1:10 dilution were 71, 86, 66, and 66, corresponding to a reduction of ookinete formation by 53% (54%), 42% (43%), 55% (56%), and 56% (56%), respectively, compared to the Trx-His (control) group (Fig. 5b). Only serum raised against PSOP25 + PSOP26 and PSOP25-PSOP26 provided significantly improved TBA versus the Trx-His group at 1:5 dilution. (Kruskal–Wallis test followed by Dunn’s multiple comparisons test, *P* < 0.05, Fig. 5b). At a 1:10 dilution, only the antisera against PSOP25 + PSOP26 significantly reduced ookinete numbers by 56% compared to the control group (Kruskal–Wallis test followed by Dunn’s multiple comparisons test, *P* < 0.05, Fig. 5b). No significant difference between anti-PSOP25-PSOP26 sera and anti-PSOP25 + PSOP26 sera was observed (Fig. 5B). Together, these results showed that the sera against mixed and fused antigens of PSOP25 and PSOP26 produced stronger inhibition effects on in vitro ookinete formation than antisera against the single PSOP25 or PSOP26 antigen.

To further examine the transmission-blocking effect of antisera recognizing PSOP25, PSOP26, mixed, or fused antigens in vivo, mice immunized with the respective recombinant antigens were infected with *P. berghei* and used for DFA. Ten days after feeding, mosquitoes were dissected and midgut oocysts were counted. The midgut oocyst densities for all immunization groups were significantly reduced by 52–66%, compared to the Trx-His immunized control (*P* < 0.001, Kruskal–Wallis test followed by Dunn’s multiple comparisons test; Fig. 5c and Table 2). There were significant reductions in oocyst density in the mixed-antigen (66%) and fused-antigen (66%) immunization groups compared with the PSOP26 (52%) immunization group (Kruskal–Wallis test followed by Dunn’s multiple comparisons test, *P* < 0.05; Fig. 5c and Table 2). The mean prevalence of mosquito infection in all immunization groups was significantly reduced by 6.7–14.5%, compared to the Trx-His immunized group (*P* < 0.05 for PSOP26; *P* < 0.01 for PSOP25, PSOP25-PSOP26, and PSOP25 + PSOP26 group; Fisher’s exact test, Fig. 5c, Table 2). However, the infection prevalence in mixed-antigen (82%) and fused-antigen (81%) immunization groups were comparable to PSOP25 (83%) or the PSOP26 (89%) immunization groups (Fig. 5d). Collectively, the mosquito feeding assays demonstrated that the ookinete surface antigens PSOP25 and PSOP26, when mixed or fused for immunization, produced significantly higher TRA than when PSOP26 was used individually; whereas PSOP25 alone could induce a promising TRA, comparable to the mixed or fusion antigens.

**Table 2:** Transmission-blocking activity of mixed or fused PSOP25 and PSOP26 antigen-immunized groups

Immunization groups	Oocyst density mean (range) (*n* = 180)	TRA^a^ (%)	Prevalence of infection mean (*n* = 180)	TBA ^b^ (%)
Control	130.6 (0–254)		94.4% (170/180)	
Trx-His	129.4 (0–254)		95.6% (172/180)	
PSOP25	48.7 (0–167)	62.3	82.8% (149/180)	12.8
PSOP26	61.5 (0–187)	52.4	88.9% (160/180)	6.7
PSOP25 + PSOP26	44.4 (0–168)	65.7	81.7% (147/180)	13.9
PSOP25-PSOP26	44.0 (0–136)	66.0	81.1% (146/180)	14.5

^a^TRA was calculated as (mean oocyst density_Trx-His_ − mean oocyst density_PSOP25/PSOP26/PSOP25+PSOP26/PSOP25-PSOP26_)/mean oocyst density_Trx-His_ × 100%

^b^TBA was calculated as % prevalence_Trx-His_ − % prevalence_PSOP25/PSOP26/PSOP25+PSOP26/PSOP25-PSOP26_

## Discussion

Here we have identified the *P. berghei* PSOP26 protein as an important factor in the zygote-to-ookinete developmental transition. We demonstrated that *psop26* transcription begins in schizonts and the expressed PSOP26 protein localizes on the surface of gametes and ookinetes, comparable to our previous report [6]. While the pre-fertilization life cycle stages are not affected in *psop26*-null mutants (Δ*psop26*), a strong reduction (> 69%) in mature ookinete numbers was observed, consistent with our report that antisera against PSOP26 could inhibit ookinete maturation [22]. However, Ukegbu et al. reported that PSOP26 is not essential for ookinete differentiation [8]. Thus, to validate the specificity of our results, we counted the zygote numbers in Δ*psop26* at 2 h after incubating at 19 °C, and found comparable zygote numbers in Δ*psop26* and WT; confirming that the reduced matured ookinete number observed in Δ*psop26* mutants is indeed caused by an impairment of the zygote-to-ookinete developmental transition. We further observed evidence of Δ*psop26* mutants having a reduction in oocyst formation, indicating that the ookinete-to-oocyst transition is also impaired. Numerous ookinete proteins have been shown to be involved in the ookinete-to-oocyst transition; including proteins which function in ookinete motility (CTRP [31], PPKL [32], CDPK3 [33], DHHC3 [34], and the IMC1 family members [35]), the *Plasmodium* CHT1 proteins involved in ookinete penetration of the midgut epithelium [36–39], and ookinete invasion-related proteins (SOAP [3], PIMMS2 [7], PSOP1/2/7/9 [5, 40], P25/P28 [2], MAOP proteins [41], and PPLP5 [42]). One report indicated that *psop26* disruption does not adversely affect ookinete motility, but mosquito midgut invasion ability was impaired [8]; suggesting that PSOP26 is an ookinete invasion-related protein. However, the function of PSOP26 during ookinete invasion remains unclear. The mosquito midgut is proposed to be an immune-competent organ, which could generate several antibacterial peptide defensins against *P. berghei* infection [43]. Thus, additional experiments will be necessary to rule out the impact of these mosquito immune factors on the function of PSOP26 protein during ookinete invasion. Taken together, these data indicate that PSOP26 is a promising target for the intervention of malaria parasite transmission.

Antigens expressed during the post-fertilization stages commonly show less amino acid polymorphism than blood-stage antigens, as they are not subjected to selective pressure by an adaptive immune response that might drive diversity. Parasites in the mosquito midgut are exposed to immune factors taken in with the vertebrate host blood, and thus are vulnerable points to disrupt the transmission cycle of the malaria parasite [44, 45]. Our previous study demonstrated that antisera against PSOP26 exhibited a moderate TBA [22]. It has been proposed that TBVs must induce sustained high antibody titers of high-affinity binding antibodies to possess high TBA [46]. Thus, to attempt to improve the TBA in the current study, we generated a chimera PSOP25-PSOP26 protein. One concern when generating multivalent vaccines is that the immunodominant component could compromise the immune response against an accompanying antigen [47–49]. To investigate this, we tested the specific antibody responses to both PSOP25 and PSOP26 recombinant proteins by ELISA analysis. We found that the antibody responses were not affected when the two antigens were presented either as a PSOP25-PSOP26 chimera protein or an equal amount mixture (PSOP25 + PSOP26). The antibody titer raised against PSOP25-PSOP26 and PSOP25 + PSOP26 antigens was not significantly different from mice that received a prime-boost with PSOP25 or PSOP26 alone. Together, these results suggest that the combination of two malaria parasite ookinete antigens did not result in substantial immune interference.

Direct feeding assays allow the assessment of TBA to determine the functionality of antibodies elicited by vaccination, and are considered the best proxy for evaluating TBV candidates [50]. Our rabbit antisera raised against both PSOP25 + PSOP26 and PSOP25-PSOP26 had significantly higher TBA, as shown by the lower percentages of infected mosquitoes and the number of oocysts per mosquito midgut, compared with PSOP26 single immunized sera and pre-immune sera. However, the antisera against fusion and mixed antigens did not exhibit significantly improved TBA compared to antisera against PSOP25 alone. One explanation is that PSOP25 is dominantly expressed on the surface of the ookinete, compared to PSOP26, as shown by our Western blot observation that antisera detected a thicker band for PSOP25 compared to PSOP26. Alternatively, PSOP25 may play a more essential role during the ookinete-to-oocyst transition, which is a hypothesis for further analysis. These results highlight the potential of selecting PSOP25 as a TBV candidate that targets the ookinete-to-oocyst developmental transition.

## Conclusions

Our study expands the role of the ookinete surface protein PSOP26 in malaria parasite transmission, and specifically demonstrates that PSOP26 has a clear and vital role in the *P. berghei* zygote-to-ookinete developmental transition. In addition, the usage of antisera against two ookinete surface antigens in the form of both fusion (PSOP25-PSOP26) and mixed (PSOP25 + PSOP26) proteins improved TBA compared to antisera against PSOP26 alone, but was comparable to the TBA antisera against PSOP25 alone. Our results confirm the previous finding that PSOP25 is a promising ookinete stage TBV candidate, and additionally found that PSOP25 alone is as good as the combination vaccine in inhibiting the ookinete-to-oocyst developmental transition. Furthermore, our data support the continued development of antisera (or antibody) against multi-stage antigens as malaria vaccine candidates to address the need for effective vaccines for the intervention of malaria parasite transmission.

## Supplementary Information

**Additional file 1: Table S1.** Primer information and sequences.

**Additional file 2: Figure S1.** Generation of HA-tagged PSOP26 transgenic parasites in the *P. berghei* ANKA line. **a** Schematic representation of the *posp26* locus (*psop26::ha*) HA tagging by double-crossover homologous recombination. The primers used for diagnostic PCR are indicated by black arrows. **b** Diagnostic PCR analysis of PSOP26::HA transgenic parasites. PCR analysis was performed using genomic DNA extracts from wild-type *P. berghei* (WT) and PSOP26::HA transgenic parasites. The native locus was detected using primers p1 + p2 (lane 1: WT, 1471 bp; PSOP26::HA, null). The 5′ and 3′ integration of a modified locus (*psop26::ha*) was detected using primers p1 + p3 (lane 2: WT, null; PSOP26::HA, 1272 bp) and p4 + p5 (lane 3: WT, null; PSOP26::HA, 1815 bp), respectively. **c** Schematic representation of *psop26* locus disruption by double-crossover homologous recombination. Primers used to detect either the WT locus or the replaced locus are marked. **d** PCR analysis of the genomic DNA from the WT and *∆psop26* parasite. Predicted DNA fragment sizes: lane 1, QCR1 + QCR2 (525 bp from WT only); lane 2, QCR2 + GW2 (753 bp from *∆psop26* only); lane 3, GW1 + GT (3500 bp from *∆psop26* only). **e** Western blot analysis shows the deletion of PSOP26. Lysates extracted from WT and Δ*psop26*-C1 parasites were incubated with α-PSOP26 or α-Hsp70. Non-infected erythrocytes (EC) were used as a negative control. PSOP26 protein bands are indicated with arrows.

**Additional file 3: Figure S2.** Giemsa staining of purified parasites. Image showing schizonts, gametocytes, zygotes, and ookinetes of the PSOP26::HA parasites.

## Data Availability

The data supporting the conclusions of this article are included within the article.

## Abbreviations

SOAP: Secreted ookinete adhesive protein
PSOPs: Putative secreted ookinete proteins
PPLP: *Plasmodium* perforin-like protein
TBVs: Transmission-blocking vaccines
TRA: Transmission-reducing activity
*An. stephensi*: *Anopheles stephensi*
nt: Nucleotide
gDNA: Genomic DNA
UTR: Untranslated region
WT: Wild-type
SDS-PAGE: Sodium dodecyl sulfate–polyacrylamide gel electrophoresis
ELISA: Enzyme-linked immunosorbent assay
PHZ: Phenylhydrazine
DFA: Direct mosquito feeding assay
*hDHFR*:*yFCU*: Human dihydrofolate reductase:yeast cytosine deaminase and uridyl-phosphoribosyltransferase
