# Supplementary material for: Characterization of PSOP26 as an ookinete surface antigen with improved transmission-blocking activity when fused with PSOP25
Source: Parasit Vectors. 2022 May 23;15:175. doi: 10.1186/s13071-022-05294-8 (PMC9125894; doi:10.1186/s13071-022-05294-8)
Supplement: Supplementary file 1 — Additional file 1: Table S1. Primer information and sequences. [file 13071_2022_5294_MOESM1_ESM.docx]

**Table S1. Primers information and sequences.**

Purpose Primer Sequence

Identification of . . ^.^QCR1 5’-AATTAGGGCTGTTTTGGGGG-3’

*∆psop26* parasites ^.^ QCR2 5’-TCAAGCTGCTAATAGACACCT-3’

**.** GW1 5’-CATACTAGCCATTTTATGTG-3’

**.** GW2 5’-CTTTGGTGACAGATACTAC-3’

**.** GT ^.^ 5’-TCACGCCGTTTGATCAGGTT-3’

Generation and identification of ^...^5UTR-F ^.^5’-cgGGGCCCGCAGGCTGTTTCGTATGTTGT-3’

PSOP26-HA parasites ^..^5UTR-R ..5’-tccCCGCGGATAGGTTCTTTTATTGCTCATC-3’

**.**3UTR-F ^.^5’-ccGGTACCAATAATCCATGTATTACATAATG-3’

**.**3UTR-R 5’-cgGAATTCCCCAACTAATGACGATTGCACTTT-3’

**.**P1 5’-GAGGAAAGACAACCAACAGCAC-3’

**.**P2 5’-TGCTGGATAAATTGCCTGCT-3’

...P3 5’-CTGGTGCTTTGAGGGGTGAG-3’

**.**P4 5’-GGTCACAAGCGGCATTCCTA-3’

**.**P5 5’-TCGATTATGGGAGCCAAAGGA-3’

RT-PCR quantification of *psop26*-F .5’-CGTGGAAATAAAGGCGATGC-3’

Expression *psop26*-R .5’-TGCTGTTGGTTGTCTTTCCT-3’

*β-tubulin*-F .5’-GGACACTATACCGAAGGTGC-3’

*β-tubulin*-R ^.^5’-CAGAAACTTTTGGCGATGGG-3’
