# Supplementary figures and images for: Characterization of PSOP26 as an ookinete surface antigen with improved transmission-blocking activity when fused with PSOP25
Source: Parasit Vectors. 2022 May 23;15:175. doi: 10.1186/s13071-022-05294-8 (PMC9125894; doi:10.1186/s13071-022-05294-8)

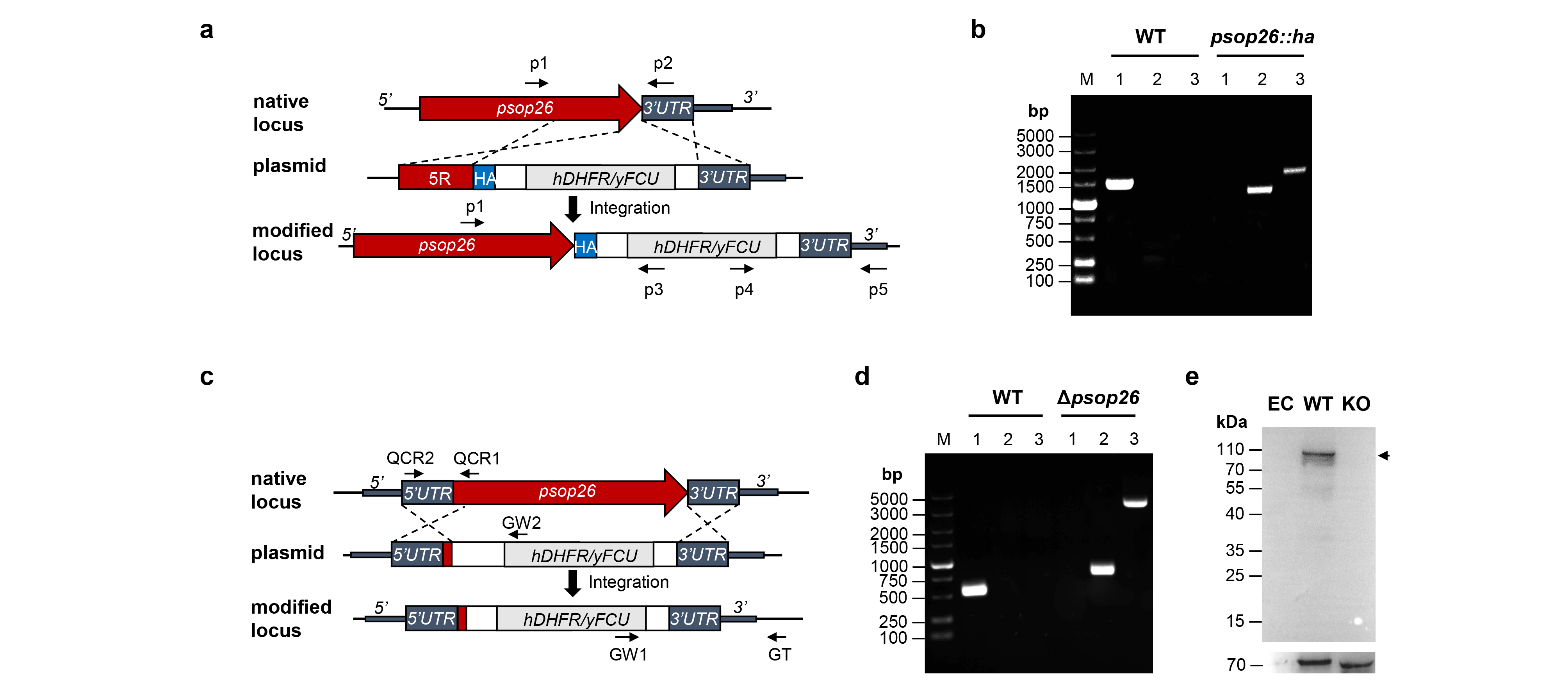

Supplement: Supplementary file 2 — Additional file 2: Figure S1. Generation of HA-tagged PSOP26 transgenic parasites in the P. berghei ANKA line. a Schematic representation of the posp26 locus (psop26::ha) HA tagging by double-crossover homologous recombination. The primers used for diagnostic PCR are indicated by black arrows. b Diagnostic PCR analysis of PSOP26::HA transgenic parasites. PCR analysis was performed using genomic DNA extracts from wild-type P. berghei (WT) and PSOP26::HA transgenic parasites. The native locus was detected using primers p1 + p2 (lane 1: WT, 1471 bp; PSOP26::HA, null). The 5′ and 3′ integration of a modified locus (psop26::ha) was detected using primers p1 + p3 (lane 2: WT, null; PSOP26::HA, 1272 bp) and p4 + p5 (lane 3: WT, null; PSOP26::HA, 1815 bp), respectively. c Schematic representation of psop26 locus disruption by double-crossover homologous recombination. Primers used to detect either the WT locus or the replaced locus are marked. d PCR analysis of the genomic DNA from the WT and ∆psop26 parasite. Predicted DNA fragment sizes: lane 1, QCR1 + QCR2 (525 bp from WT only); lane 2, QCR2 + GW2 (753 bp from ∆psop26 only); lane 3, GW1 + GT (3500 bp from ∆psop26 only). e Western blot analysis shows the deletion of PSOP26. Lysates extracted from WT and Δpsop26-C1 parasites were incubated with α-PSOP26 or α-Hsp70. Non-infected erythrocytes (EC) were used as a negative control. PSOP26 protein bands are indicated with arrows. [file 13071_2022_5294_MOESM2_ESM.tif]

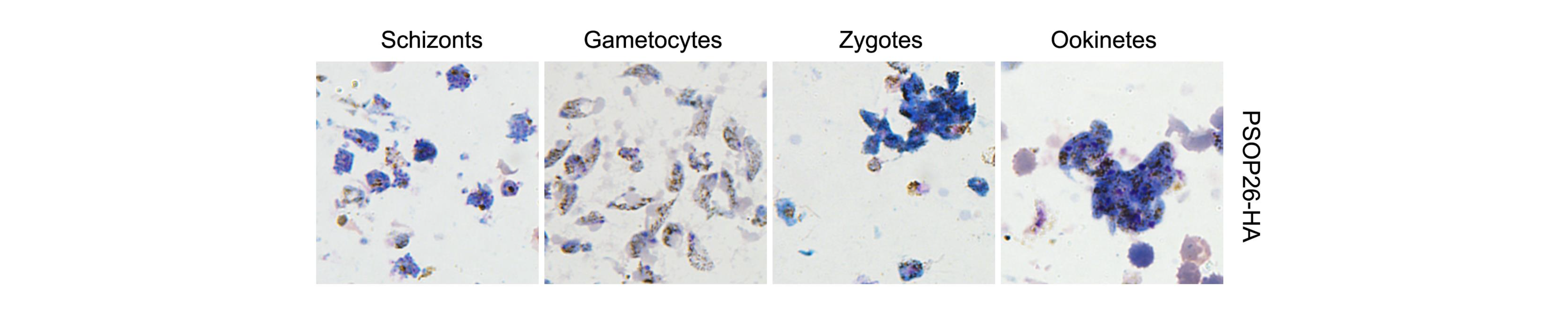

Supplement: Supplementary file 3 — Additional file 3: Figure S2. Giemsa staining of purified parasites. Image showing schizonts, gametocytes, zygotes, and ookinetes of the PSOP26::HA parasites. [file 13071_2022_5294_MOESM3_ESM.tif]
